# Cytokine patterns in a prospective cohort of HIV-infected patients with cryptococcal meningitis following initiation of antifungal and antiretroviral therapy

**DOI:** 10.1371/journal.pone.0176304

**Published:** 2017-05-09

**Authors:** Delio José Mora, Kennio Ferreira-Paim, Leonardo Eurípedes Andrade-Silva, Thatiane Bragine, Ivonete Helena Rocha, Barbara de Melo Ribeiro, Guilherme Henrique Machado, Virmondes Rodrigues Junior, David Nascimento Silva-Teixeira, Wieland Meyer, Mario León Silva-Vergara

**Affiliations:** 1 Infectious Diseases Unit, Triângulo Mineiro Federal University, Uberaba, Minas Gerais, Brazil; 2 Laboratory of Immunology, Triângulo Mineiro Federal University, Uberaba, Minas Gerais, Brazil; 3 Institute of Health Sciences, Department of Clinical Medicine, Triângulo Mineiro Federal University, Uberaba, Minas Gerais, Brazil; 4 Molecular Mycology Research Laboratory, Centre for Infectious Diseases and Microbiology, Marie Bashir Institute for Emerging Infectious Diseases and Biosecurity, Sydney Medical School-Westmead Hospital, The Westmead Institute for Medical Research, The University of Sydney, Sydney, Australia; University of Birmingham, UNITED KINGDOM

## Abstract

Cryptococcal meningitis (CM) is a life-threatening infection in HIV-infected patients, especially in resource-limited settings. Cytokine patterns in the cerebrospinal fluid (CSF) and sera may be related to clinical outcomes. This study aimed to evaluate cytokine patterns in the CSF and sera of HIV-infected patients with CM as well as the cytokines produced by peripheral blood mononuclear cells (PBMCs) when stimulated with LPS and cryptococcal GXM. CSF and serum levels of IL-2, IL-4, IL-8, IL-10, IL-12p40, IL-17A, INF-γ, TNF-α and CXCL-10 were measured in HIV-infected patients with CM (CM^+^ HIV^+^) at various time points. Cytokine levels were evaluated in the PBMC culture supernatants and the baseline values were compared to those of HIV-infected patients without CM (CM^-^ HIV^+^) and healthy controls (CM^-^ HIV^-^). CSF cytokine levels at admission (n = 33) were higher than levels among the 23 survivors at week 2, but statistically significant differences were observed for IL-8 and IFN-γ (p<0.05). CSF and serum levels of IL-4 and IL-17A at week 10 (n = 16) were lower than the baseline values, whereas IL-2 levels increased compared to week 2 (p<0.05). At week 16 (n = 15), CSF and serum levels of IL-4, IL-10 and CXCL-10 were decreased compared to the baseline values (p<0.05). PBMCs from CM^-^ HIV^-^ individuals produced significantly higher levels of proinflammatory cytokines in response to LPS, with the exception of TNF-α, which showed higher levels among CM^+^ HIV^+^ patients. The PBMCs of CM patients produced higher levels of IL-4 than those of CM^-^ HIV^-^ patients in response to GXM stimulation, and levels progressively decreased during treatment (p<0.05). Then, a progressive shift in cytokine expression favoring a Th1 pattern was observed, which is crucial in controlling cryptococcal infection. A better understanding of the protective immune response against *Cryptococcus neoformans* will help to develop novel strategies to improve the outcomes of patients with cryptococcosis.

## Introduction

The global Human Immunodeficiency Virus (HIV) pandemic has led to a dramatic increase in cryptococcosis cases during the past several decades. It is estimated that 957,900 cases of cryptococcal meningitis (CM) associated with HIV infection occur globally every year, of which 720,000 occur in sub-Saharan Africa [1]. Despite progressive increases in access to antiretroviral therapy (ART), cryptococcal infection continues to occur in patients with late HIV diagnosis and CD4^+^ T cell count < 100 cells/μL at admission, and it is responsible for >40% of AIDS-attributable mortality in resource-limited settings [2–4].

A healthy immune response against cryptococcal infection in immunocompetent hosts depends on coordinated interactions between antigen-presenting cells (APCs) and effector T cells to generate a robust cellular immune response [5]. Type-1 helper T cell (Th1) and Th2 cytokines respond to *Cryptococcus* differently. Th1 elicits IL-6, IL-8, IL1-β, IFN-γ and TNF-α production to recruit and activate macrophages and up-regulate the production of reactive oxygen species to kill invading fungi, thus initiating a specific adaptive CD4^+^ T-cell response [6,7,4]. In contrast, IL-4, IL-10 and IL-13 act as downregulators of the cellular immune response, inhibiting T-cell proliferation, and are associated with impaired infection control and a poor outcome [8,9]. Macrophages also release granulocyte colony-stimulating factor (G-CSF), which up-regulates leukotriene synthesis and the anticryptococcal activity of neutrophils [10]. In addition, T-helper 17 cytokines have been associated with increased macrophage activation, more rapid clearance of *Cryptococcus* from the CSF, and improved 2-week survival [9,11–13].

HIV-infected patients with CM show a minimal proinflammatory immune response and a high fungal and antigenic burden in the cerebrospinal fluid (CSF) at admission, which may be associated with adverse clinical and microbiological outcomes [14,15]. Glucuronoxylomannan (GXM), a major cryptococcal polysaccharide capsule antigen, activates APCs via toll-like receptor 4 and CD14 in healthy individuals but, in HIV-infected patients, it is associated with fungal persistence and pathogenicity, as it allows fungal cells to evade and overwhelm the immune system [16,17]. This virulence factor interacts with macrophages, decreasing their antiphagocytic properties, and interferes with T-cell function and proliferation, which results in disseminated fungal infection [18,19].

Host immune responses against *C*. *neoformans* are complex and need to be evaluated by various methods to elucidate the mechanisms underlying the pathogenesis of CM and improve the survival of these patients. This study aimed to assess cytokine patterns in HIV-infected patients with CM at several time points during antifungal and antiretroviral therapy in a Brazilian hospital.

## Methods

### Subjects

This study was carried out in the Infectious Diseases Unit of the Teaching Hospital at Triângulo Mineiro Federal University in Uberaba, Minas Gerais State, Brazil, from August 2008 to November 2014. Thirty-three out of 42 HIV-infected patients aged ≥ 18 years who presented with cryptococcal meningitis were prospectively enrolled and followed-up. The remaining nine patients were severely ill when they were admitted and died before being diagnosed with cryptococcal infection and/or treatment initiation. HIV infection was diagnosed according to the recommendations of the Brazilian Ministry of Health, on the basis of 2 positive enzyme-linked immunoassay (ELISA) tests plus a positive Western blot, immunofluorescence or PCR confirmatory test [20].

Cases of cryptococcal meningitis were defined based on clinical picture and laboratory features, including positive CSF India ink staining, a positive cryptococcal antigen (CrAg) test, and positive CSF *C*. *neoformans* cultures. Epidemiological, clinical, laboratory and outcome data were obtained from the medical records. Patients with CM received induction therapy with amphotericin B (0.7–1.0 mg/Kg/day) alone or in combination with fluconazole (400 mg 2x/day) for two weeks, followed by consolidation therapy with fluconazole (400 mg 2x/day) for eight weeks, and maintenance therapy with fluconazole (200 mg/day). ART was started 2–4 weeks after CM diagnosis, in accordance with the clinical practice guidelines for the management of cryptococcal disease of the Infectious Diseases Society of America, which recommend an interval of 2–10 weeks [21].

CSF, serum and PBMC culture supernatants were collected and assessed prospectively, at diagnosis (baseline), after induction therapy (week 2), at the end of consolidation therapy (week 10) and at week 16 (to evaluate the effect of ART). All patients underwent CD4^+^ T cell count and viral load tests at admission and at week 16. The control group for the baseline PBMC cytokine assessment consisted of 56 HIV-positive individuals without CM (CM^-^ HIV^+^), matched by CD4^+^ T-cell count, age and gender, who were admitted to the hospital for other infections (e.g., toxoplasmosis, Chagas disease, syphilis, tuberculosis, cytomegalovirus (CMV) diseases and Paracoccidioidomycosis) or non-infectious neurological symptoms (epilepsy, migraine, stroke, dementia, tension-type headache, etc.). A second control group included 48 HIV-negative patients without cryptococcosis (CM^-^ HIV^-^) who were admitted to the emergency room for a variety of reasons, including migraine, epilepsy, tension-type headache, stroke, and dementia. A detailed description of these patients was previously published [13].

### Laboratory assessment

Both CSF and blood samples were obtained from CM patients at admission and at various times during follow-up. After lumbar puncture, 6 mL of CSF were collected and separated into two tubes. The first tube was used to perform a cell count, biochemical analyses, CrAg titers and quantitative fungal culture. The second tube was aliquoted (500 μL/vial) and stored at -70°C for subsequent cytokine assays. Additionally, 10 mL of peripheral venous blood were obtained and centrifuged (2500 rpm), and the serum was aliquoted (500 μL/vial) and stored at -70°C for subsequent cytokine assays and CrAg quantification. Aliquots were coded with the patient’s protocol number. The CSF and serum CrAg titers were measured using the latex detection system and evaluated based on the agglutination observed in serial dilutions (IMMY Mycologics Inc., OK, USA). Quantitative fungal culture was performed as described elsewhere [22].

### Isolation and stimulation of PBMCs

Heparinized venous blood was diluted with RPMI 1640 medium (SIGMA, USA) plus 5% fetal bovine serum, and the mononuclear cell layer were separated by density gradient centrifugation at 2500 rpm (Sorvall-Legend Mach 1.6R, Germany) on Ficoll-Hypaque (GE Healthcare, Sweden). The mononuclear cell layers were washed three times in RPMI 1640, and then resuspended in complete medium (cRPMI: RPMI 1640 supplemented with 1% L-glutamine, 1% HEPES, 1% Penicillin-Streptomycin and 5% fetal bovine serum) and incubated for 24 h on flat-bottomed 24-well tissue culture plates (Techno Plastic Products, Trasadingen, Switzerland) at 37°C and 5% CO_2_ (SANYO, Japan). Cellular viability > 95% was verified using trypan blue dye.

The capsular polysaccharide (GXM) of *C*. *neoformans* was obtained from the supernatant of serotype A (ATCC 90112) cultures using CTAB-GXM precipitation and identified using the Cryptolatex test and GXM-specific monoclonal antibody 18b7 [23,24]. Isolated PBMCs (2 x 10^6^ cells/mL) were stimulated with 10 μg/mL of lipopolysaccharide (LPS) from *Escherichia coli* 026:B6 (SIGMA-USA) and 10 μg/mL of GXM for 48 h on flat-bottomed 24-well tissue culture plates (Techno Plastic Products, Trasadingen, Switzerland) at 37°C and 5% CO_2_ (SANYO, Japan). PBMC negative controls were incubated with RPMI 1640 only. After incubation, PBMC culture supernatants were harvested, frozen, and stored at -70°C for subsequent cytokine assays.

### Cytokine assays

Cytokines in the serum, CSF and PBMC culture supernatants were measured using a sandwich enzyme-linked immunosorbent assay (ELISA). Tumor necrosis factor-α (TNF-α), interferon-γ (INF-γ), interleukin-2 (IL-2), IL-4, IL-8, IL-10, IL-12p40 (Becton Dickinson, USA), IL-17A and CXCL-10 (R&D Systems, USA) were quantified in duplicate using a luminometer (Turner Biosystems, Sunnyvale, CA, USA), according to the manufacturer’s instructions [13,25]. The results were expressed in picograms per milliliter based on a standard curve.

### Ethics statement

The study design and protocol were approved by the Research Ethics Board of the Triângulo Mineiro Federal University under protocol #1350. Written informed consent was obtained from each participant. The Strengthening the Reporting of Observational Studies in Epidemiology (STROBE) guidelines were followed in reporting this observational study [26].

### Statistical analysis

Categorical variables were analyzed using the χ^2^ test or Fisher’s exact test. Continuous variables with a normal distribution were analyzed using Student’s t-test, and continuous variables with non-normal distributions were analyzed using the Mann-Whitney U-test. Median cytokine levels were compared using the Mann-Whitney U-test or the Kruskal-Wallis test with Dunn's multiple comparison test, depending on the number of groups being compared. Statistical analyses were performed using MedCalc for Windows version 11.3 (MedCalc Software, Ostend, Belgium) and GraphPad Prism v6 (GraphPad software Inc, CA USA). For all tests, p values < 0.05 were considered statistically significant.

## Results

### Baseline epidemiological and laboratory data

Of 33 HIV-infected patients with CM, 27 (81.8%) were male, and the mean age was 37.4 years (interquartile range [IQR], 23–55). Cryptococcal meningitis was the first AIDS-defining condition in 20 (60.6%) cases, and in 13 (65%) cases, both diseases were diagnosed simultaneously at admission. The median CD4^+^ T cell count was 37/μL (IQR, 19–81) and the median HIV viral load was 5.2 log_10_ RNA copies/mL (IQR, 4.5–6.1). The most prevalent risk factor for HIV infection was unprotected heterosexual intercourse, which was reported by 28 (84.8%) individuals, 14 (50%) of whom used illicit drugs as well. The cumulative incidence of mortality was 30.3% (10/33) at week 2, 51.5% (17/33) at week 10, and 54.5% (18/33) at the end of follow-up. Four patients died before starting antifungal therapy. Currently, nine out of 15 survival patients at week 2 were still alive at week 16. No cases of immune reconstitution inflammatory syndrome (IRIS) were diagnosed during the study period.

### Laboratory assessment

The median CD4^+^ T cell count at week 16 (89 cells/μL [IQR, 79–152]) showed a 2.4-fold increase compared with the baseline value (p>0.05). At week 16, the HIV viral load values (1.8 log_10_ RNA copies/mL [IQR, 1.2–3.1]) were significantly lower than those observed at baseline (p = 0.045). The prospective assessment of CSF showed that the median CSF white blood cell (WBC) count at week 10 (28 cells/μL [IQR, 14–139], with lymphocytic predominance) increased 1.8-fold compared to baseline (15 cells/μL [IQR, 1–114]) (p>0.05). By week 2, CSF cryptococcal colony-forming unit counts had decreased rapidly, from a median of 5.32 log_10_ CFU/mL (IQR, 3.76–5.9) at baseline to 1.07 log_10_ CFU/mL (IQR, 0–2.1) in 5/20 (25%) positive cultures (p<0.05).

The CrAg titers decreased more slowly, from a median of 1024 (IQR, 512–4096) at baseline to a median of 256 (IQR, 128–512) at week 2 and 128 (IQR, 32–128) at week 10. The rate of decline of cryptococcal CFU counts was not correlated with that of CSF CrAg titers (Pearson’s r = 0.17 [95% CI,—0.14 to 0.46]; p = 0.48). The CSF protein concentration decreased, from a median of 0.78 g/dL (IQR, 0.42–1.5) at baseline to 0.56 g/dL (IQR, 0.38–0.73) at week 10 (p = 0.47). The CSF glucose concentration increased from a median of 27.9 mg/dL (IQR, 19–51.2) at baseline to a median of 49.3 mg/dL (IQR, 35.1–58.2; p = 0.043) (Table 1).

**Table 1:** Laboratory assessment of HIV-infected patients with cryptococcal meningitis.

Parameters	Baseline N = 33	Week 2 N = 23	Week 10 N = 16		Week 16 N = 15
CD4^+^ T-cell count, cells/μL	37 (19–81)	NA		NA	89 (79–152)
HIV load, log_10_ copies RNA/mL	5.2 (4.5–6.1)	NA		NA	1.8 (1.2–3.1)
CSF log_10_ CFU/mL	5.32 (3.76–5.9)	1.07 (0–2.1)		negative	negative
CSF CrAg titer	1024 (512–4096)	256 (128–512)		128 (32–128)	32 (8–64)
CSF WBC count, cells/μL	15 (1–114)	21 (10–118)		28 (14–139)	35 (19–157)
CSF glucose, mg/dL	27.9 (19–51.2)	37.4 (22.5–54)		49.3 (35.1–58.2)	54.1 (38.9–73.6)
CSF protein, g/dL	0.78 (0.42–1.5)	0.69 (0.39–1.1)		0.56 (0.38–0.93)	0.43 (0.34–0.73)

The data presented are medians (interquartile range, [IQR]). Abbreviations: CFU, Colony-forming units; CrAg, cryptococcal glucuronoxylomannan antigen; CSF, cerebrospinal fluid; WBC, white blood cell; NA, not available.

### Changes in cytokine levels during therapy

**Fig 1:**
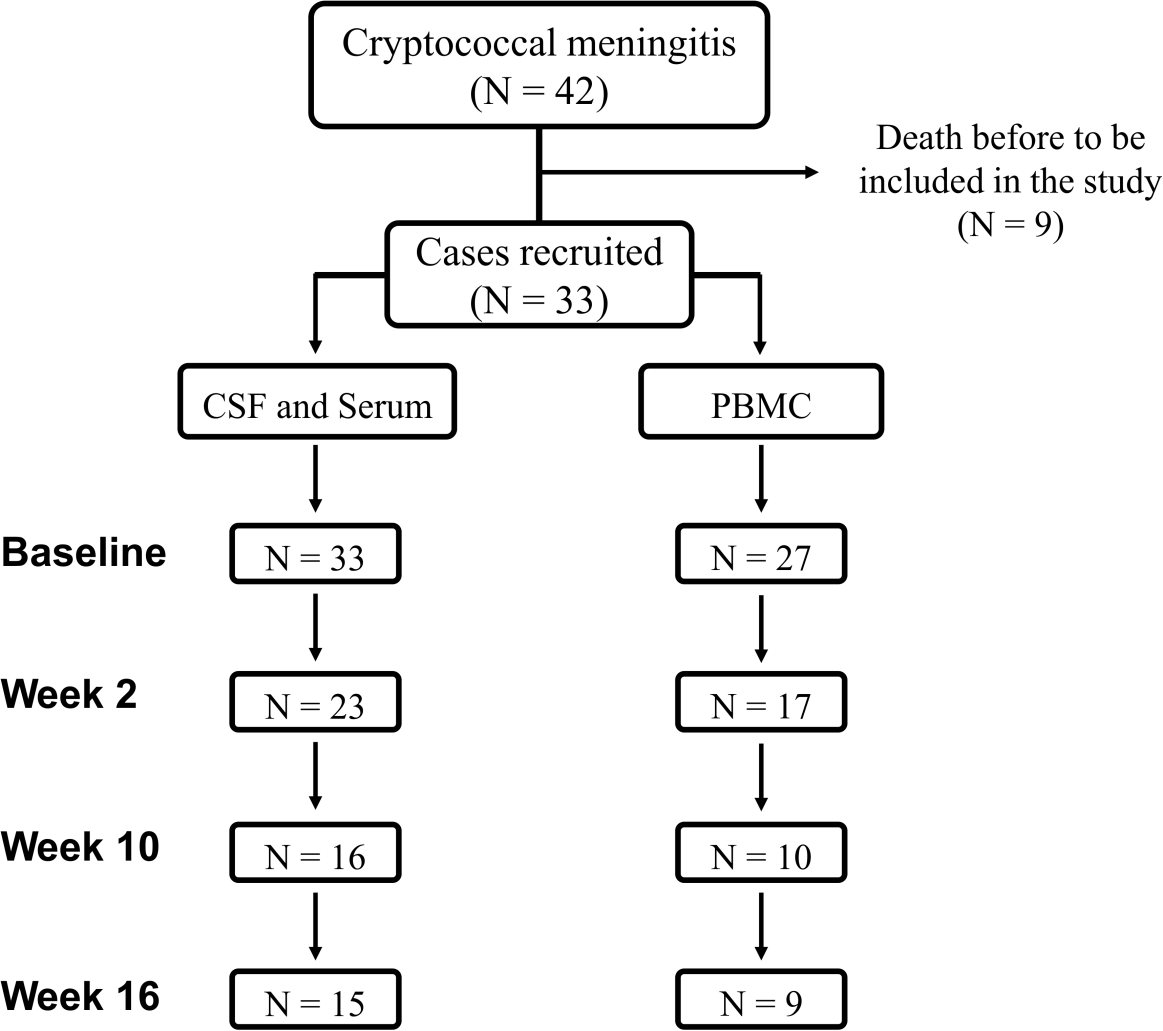
Patient flow chart. Distribution of HIV-infected patients in whom cytokine production assays were performed. Abbreviations: CSF, cerebrospinal fluid; PBMCs, peripheral blood mononuclear cells.

CSF and serum cytokine levels were measured prospectively at baseline (n = 33), at week 2 (n = 23), at week 10 (n = 16) and at week 16 (n = 15). In addition, cytokine levels in PBMCs were evaluated at baseline (n = 27), at week 2 (n = 17), at week 10 (n = 10) and at week 16 (n = 9) (Fig 1).

**Fig 2:**
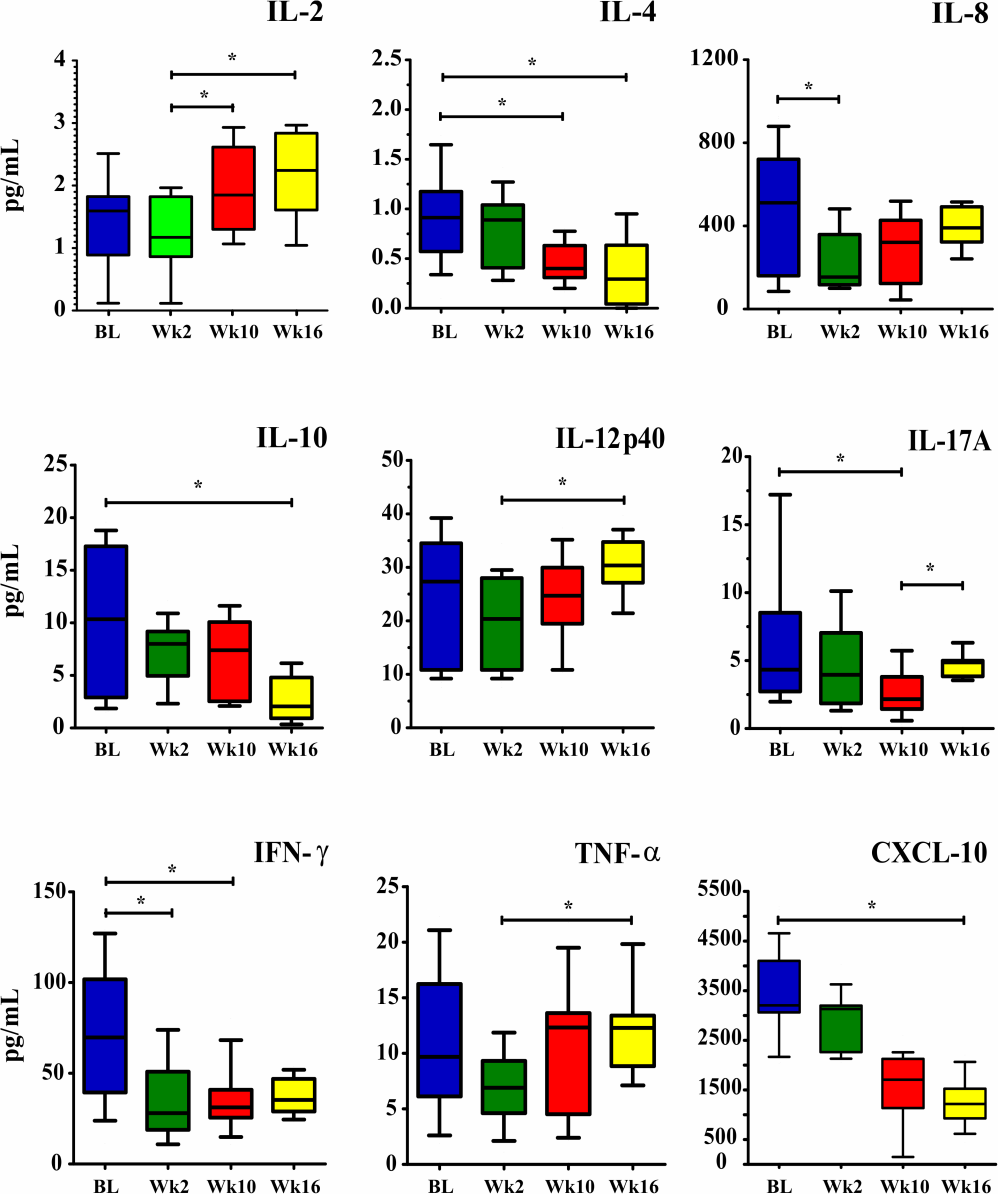
Changes in CSF cytokine levels (pg/mL) at admission (baseline), week 2, week 10 and week 16 on ART. Data are shown as boxes: internal horizontal lines, medians; top and bottom of boxes, 25th and 75th percentiles; upper and lower bars, 10th and 90th percentiles. Statistical comparisons between groups were performed using the Kruskal-Wallis test followed by Dunn’s test. The symbols (*p<0.05) reflect statistical analyses based on comparisons of the four time points. Abbreviations: BL, Baseline; Wk2, week 2; Wk10, week 10; Wk16, week 16.

The median baseline CSF cytokine levels of the 33 patients included in the study were higher than those of the 23 survivors at week 2, statistically significant differences were only observed for IL-8 and IFN-γ (p = 0.042 and 0.031, respectively). At week 10, the IL-2, IL-8, IL-12p40, IL-17A, IFN-γ and TNF-α levels in the 16 survivors were higher than the levels observed at week 2, but statistical significance was only observed for IL-2 (p = 0.047). In contrast, IL-10 levels were significantly higher among patients who died (p = 0.036). Levels of IL-4, IL-17A and IFN-γ were significantly decreased at week 10 compared to baseline (p = 0.031, 0.032 and 0.039, respectively). At week 16, IL-4, IL-10 and CXCL-10 levels were significantly decreased compared to baseline (p = 0.042, 0.039 and 0.036, respectively) (Fig 2, S1 Dataset). Five out of 23 (21.7%) patients who had positive cultures at week 2 exhibited significantly lower levels of IFN-γ and IL-17A and higher levels of IL-4 and IL-10 compared to those who had negative CSF cultures (all p<0.05).

**Fig 3:**
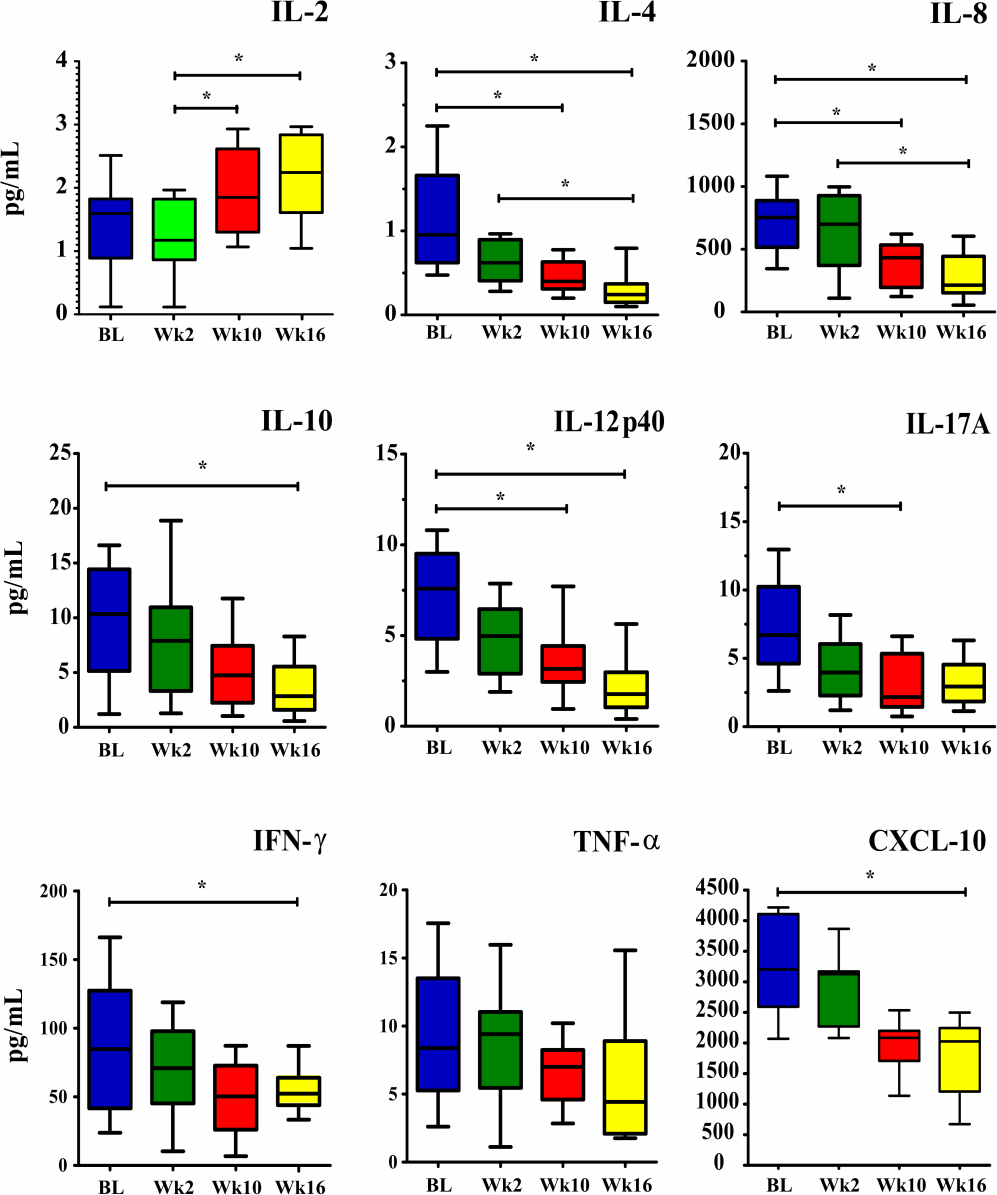
Changes in serum cytokine levels (pg/mL) at admission (baseline), week 2, week 10 and week 16 on ART. Data are shown as boxes: internal horizontal lines, median; top and bottom of boxes, 25th and 75th percentiles; upper and lower bars, 10th and 90th percentiles. Statistical comparisons between groups were performed using the Kruskal-Wallis test followed by Dunn’s test. The symbols (*p<0.05) reflect statistical analyses based on comparisons of the four time points. Abbreviations: BL, Baseline; Wk2, week 2; Wk10, week 10; Wk16, week 16.

The median of most serum cytokine levels at week 2 were lower than those at baseline (Fig 3). At week 10 median serum levels of IL-4, IL-8, IL12p40 and IL-17A were decreased compared to baseline (all p<0.05). The levels of IL-4, IL-8, IL-10, IL-12p40, IFN-γ and CXCL-10 were significantly decreased at week 16 compared to baseline (all p<0.05), S2 Dataset.

### Cytokine profiles induced by cryptococcal capsular polysaccharide (GXM) stimulation of PBMCs

**Fig 4:**
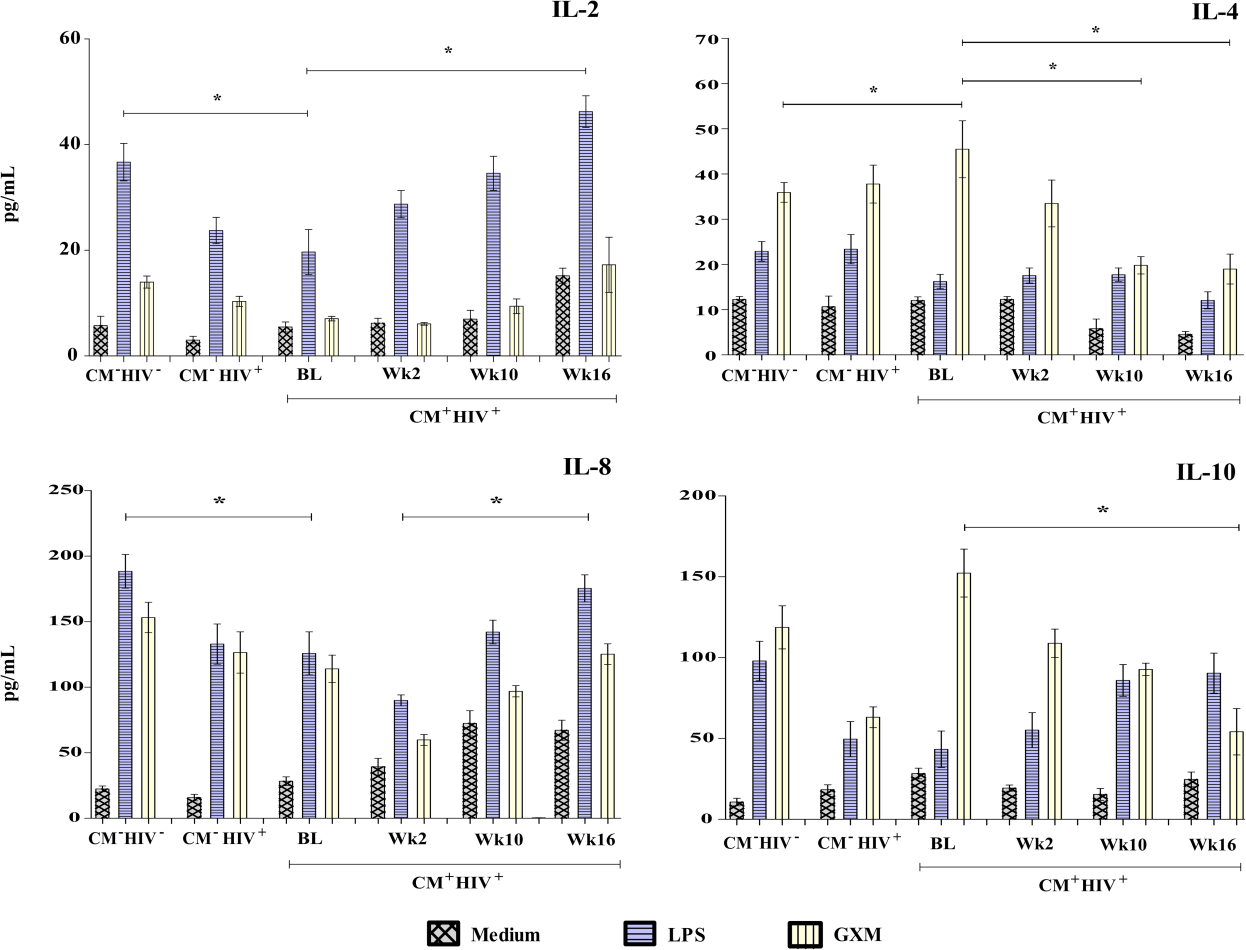
IL-2, IL-4, IL-8, and IL-10 release. PBMCs (2 x 10^6^ cells/mL) from HIV-infected patients with cryptococcal meningitis (CM^+^ HIV^+^) stimulated with 10 μg/mL of lipopolysaccharide (LPS) and 10 mg/mL of GXM for 48 h or treated with a control were evaluated at various times during treatment. Control groups: HIV patients without cryptococcosis (CM^-^ HIV^+^) and HIV-negative individuals (CM^-^ HIV^-^). Statistically significant differences are marked with * (p <0.05, Mann-Whitney test). Abbreviations: BL, Baseline; Wk2, week 2; Wk10, week 10; Wk16, week 16.

**Fig 5:**
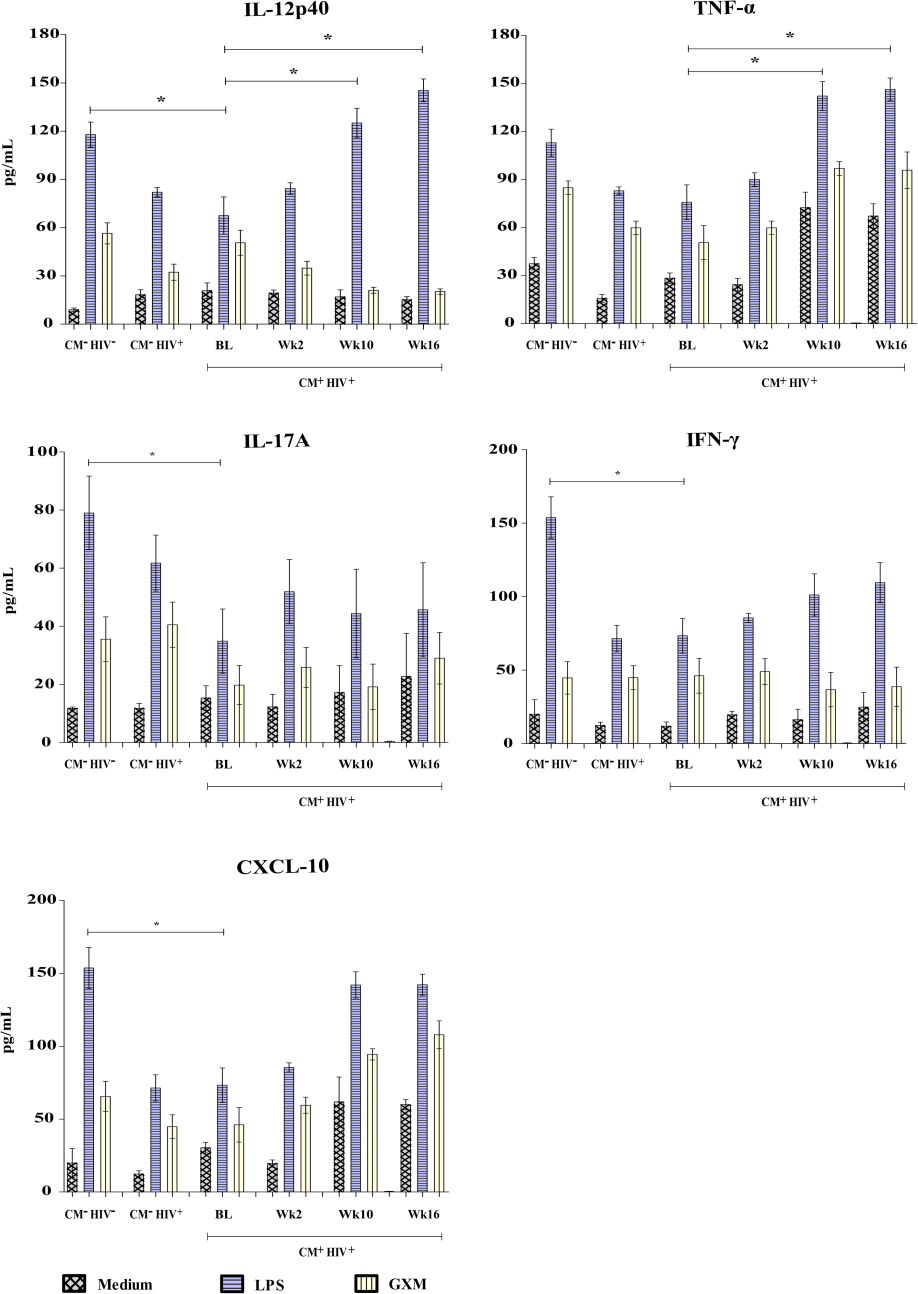
IL-12p40, TNF-α, IL-17A, IFN-γ, and CXCL-10 release. PBMCs (2 x 10^6^ cells/mL) from HIV-infected patients with cryptococcal meningitis (CM^+^ HIV^+^) stimulated with 10 μg/mL of lipopolysaccharide (LPS) and 10 mg/mL of GXM for 48 h or treated with a control were evaluated at various times during treatment. Control groups: HIV patients without cryptococcosis (CM^-^ HIV^+^) and HIV-negative individuals (CM^-^ HIV^-^). Statistically significant differences are marked with * (p <0.05, Mann-Whitney test). Abbreviations: BL, Baseline; Wk2, week 2; Wk10, week 10; Wk16, week 16.

Levels of IL-2, IL-4, IL-8, IL-10, IL-12p40, IL-17A, TNF-α, IFN-γ and CXCL-10 were measured in the PBMCs of 27 HIV-infected patients with CM after *in vitro* stimulation with GXM, before and during antifungal and antiretroviral therapy; levels in CM patients were compared with those of patients in the two control groups. At admission, the PBMCs of CM^-^ HIV^-^ individuals produced higher levels of IL-2, IL-12p40, IL-17A, IFN-γ and CXCL-10 in response to LPS than those of CM^-^ HIV^+^ patients (all p<0.05). A significant increase in levels of IL-12p40 and TNF-α in response to LPS was observed in CM^+^ HIV^+^ patients at weeks 10 and 16, compared to baseline (p = 0.047 and 0.041, respectively). The GXM-stimulated PBMCs of CM^+^ HIV^+^ patients produced higher levels of IL-4 at baseline than those of the CM^-^ HIV^-^ controls (S3 Dataset). Levels of IL-4 and IL-10 progressively decreased during follow-up (p = 0.035 and 0.046, respectively), as shown in Figs 4 and 5.

## Discussion

This study aimed to identify cytokine patterns in HIV-infected patients with CM at different times. The results obtained support previous reports that CSF cytokine patterns differ from serum cytokine patterns in HIV-infected patients with CM and that there is a change in cytokine levels during antifungal and antiretroviral therapy. High levels of pro-inflammatory cytokines and decreased levels of IL-4 and IL-10 in GXM-stimulated PBMCs were observed during antifungal and antiretroviral therapy. These findings provide additional insight into the modulation of the immune response against *C*. *neoformans*.

Currently, most cases of cryptococcal meningitis occur in HIV-infected patients and a high mortality rate is still observed in limited-resource settings in Sub-Saharan Africa, Southeast Asia and Latin America [1,2]. These poor outcomes are directly attributable to a late diagnosis, advanced immunodeficiency and severe disseminated fungal infection at presentation. According to other authors, most patients die during the first weeks on antifungal therapy, and it is possible that early interventions including optimal antifungal therapy could prevent some of these fatalities [27,28].

In Brazil, 4–6% of HIV-infected patients present with cryptococcosis at some point during their lifetime, and 55% of these patients die [29]. Cryptococcosis accounts for 50.9% of AIDS deaths due to systemic mycoses, followed by candidiasis (30.2%) and histoplasmosis (10.1%) [30]. Since 1997, ART has been freely available through public health services for HIV-infected patients, yet most patients who are diagnosed with cryptococcosis are not receiving this therapy or have poor adhesion to treatment [31,32].

This prospective study recruited 33 HIV-infected patients with CM over four years. To evaluate cytokine patterns during antifungal and antiretroviral treatment, patients were followed up for 16 weeks. At admission, most patients presented several clinical and laboratory features associated with poor outcomes, such as increased intracranial pressure, altered consciousness, high fungal burden and low CSF inflammation [13,31,33].

CSF analysis with India ink stain, fungal culture, and cytochemical and immunological tests provide relevant data that, in some ways, reflect the clinical and immune status of the host [21,34]. A high baseline CSF protein concentration is one of the most sensitive predictors of neurological disease, increased inflammatory response and high CrAg titers [34,35]. Patients in the present study who had a good outcome had higher baseline CSF protein concentrations, which suggests an increased inflammatory response in the CNS and improvement of AMB transport [36]. Moreover, glucose levels progressively increased as fungal clearance progressed, which is in line with previous reports [37,38].

Several authors have correlated the CSF CrAg baseline titers with the fungal burden, demonstrating that titers can serve as an alternative measure of the fungal load; however, this correlation becomes unclear once antifungal therapy is started [22,39]. Moreover, high CSF CrAg titers are associated with poor prognosis in patients with CM and are often associated with high ICP [40–42]. A significant positive correlation between CSF CFU counts and CSF CrAg titers at admission was observed in a Thai cohort of HIV-positive patients with CM, but the rapid rate of decline in CFU counts was not correlated with CrAg titers [22].

Similar features were observed in most cases in the present cohort, although CSF CrAg titers decreased in four patients who died early in the study, causing a misleading impression of pathogen clearance. In addition, two patients who showed clinical improvement had increased CrAg titers during antifungal therapy and their titers remained detectable after therapy even though the Nankim stain and culture turned out negative. Most patients presented a decrease in CSF CrAg titers during treatment. According to other authors, titers could be used to monitor response to antifungal therapy but not as an index of cure and they must be interpreted in the context of clinical features [43]. Thus, the discrepancy between CrAg titers and CFU counts reflects the degree of CrAg shedding by *C*. *neoformans* and is associated with the host immune response [44].

Cytokines are key modulators of the immune response and play an essential role in the defense mechanism against fungal infections [45]. Previous studies have shown the importance of pro-inflammatory responses at the infection site; for instance, IFN-γ improves host immune responses against cryptococcal infection in HIV-infected patients [46,47]. Paradoxically, the baseline levels of cytokines observed in these patients cannot control the infection due to advanced immunodeficiency, as corroborated by the low CD4 counts and low CSF white cell counts observed. The significant decrease in IL-8 and IFN-γ levels observed at week 2 could be attributed to the decrease in antigenic stimulation following antifungal therapy [9,46,48]. These findings are in line with those observed in animal models, thereby reinforcing the role of IL-8, IL-12, IFN-γ, TNF-α and CXCL10 in cryptococcal infection control [8,49].

Previous studies in patients with CM evaluated CSF cytokine levels on the third day of antifungal therapy and found elevated levels of IL-6, IL-1β, TNF-α and IFN-γ compared to baseline. The authors suggested that this finding was due to the increased antigenic stimulation caused by the shedding of capsular components from dead fungal cells, together with the immunostimulatory effect of AMB [46,48–51]. In this study, a gradual increase of pro-inflammatory cytokine levels and a decrease of IL-4 and IL-10 levels during follow-up were observed. Previous evidence suggests that IL-4 suppresses host defense mechanisms against *C*. *neoformans*, probably through the inhibition of local IFN-γ production [52]. In a recent study, low baseline CSF and serum TNF-α and IFN-γ levels in patients with CM were associated with a poor outcome at weeks 2 and 10 on therapy [13]. Moreover, adjunctive IFN-γ therapy appears to be safe and augments fungal clearance without evidence of adverse effects on HIV viral control or IRIS [47,53]. A CSF pro-inflammatory response consists of an interplay of Th1 (IFN-γ and IL‑6), Th2 (IL‑4 and IL‑10) and Th17 cytokines (IL‑17A) and has been shown to be highly predictive of increased macrophage activation, rapid clearance of infection and consequently better survival in patients with CM [9].

During cryptococcal infection, capsule components with immunosuppressive and antiphagocytic properties such as GXM, and probably GalXM and MPs, circulate in the host [54]. In the *in vitro* study reported herein, purified GXM suppressed the induction of PBMC proinflammatory cytokines before and during antifungal therapy but induced both IL-4 and IL-10. Other *in vitro* studies have shown that IL-10 directly inhibits proinflammatory cytokine production and antigen presentation by macrophages, resulting in additional impairment of IFN-γ-induced CD4^+^ T-cell production and conditions favorable for cryptococcal growth [55–57].

Moreover, GXM-stimulated PBMCs from healthy donors produced higher levels of IL-4 and IL-10 than those stimulated with LPS after 48 hours. This finding may be clinically relevant, as high antigen concentrations are frequently found in the body fluids of AIDS patients with CM and are considered one of the most important prognostic factors associated with a poor outcome [58]. In a recent study, poor survival in a cohort of AIDS patients with CM was associated with decreased monocyte production of TNF-α and IFN-γ in whole blood stimulated with LPS [59]. Other studies of patients with pulmonary cryptococcosis have reported that PBMCs incubated with recombinant IL-12 produced high levels of IFN-γ, which contributes to fungal clearance [4,60].

Several previous studies have reported the *in vitro* induction of cytokines in effector cells after stimulation with *C*. *neoformans* or its capsular components [49,60–63]. However, cytokine induction levels can vary depending on the experimental conditions [4]. Thus, it is difficult to interpret variations in the results of experiments in which one or more cytokines were induced by *C*. *neoformans*. Variables in the experimental design of cytokine induction studies include: cryptococcal strains, purification methods of the cryptococcal components and effector cells used, among others [53,60–64].

Despite the low number of CM patients included in this study, the cytokine dynamics observed over 16 weeks of follow-up reflect the impact of antifungal therapy and ART and can help predict the outcomes of these patients. Besides, the HIV-infected patients group control who presented concurrent infections may influence their immune response and therefore cytokine profile. Better knowledge of cytokine dynamics can help to develop novel strategies to improve the outcomes of patients with cryptococcosis, especially in resource-poor settings around the world.

## Supporting information

S1 Dataset Cerebrospinal fluid cytokines levels of HIV-infected patients with Cryptococcal meningitis before and during antifungal an antiretroviral therapy. (XLSX)

S2 Dataset Serum cytokines levels of HIV-infected patients with Cryptococcal meningitis before and during antifungal an antiretroviral therapy. (XLSX)

S3 Dataset Peripheral blood mononuclear cells supernatant cytokines levels of HIV-infected patients with Cryptococcal meningitis before and during antifungal an antiretroviral therapy. (XLSX)
